# Analysis of cumulative outcomes and influencing factors of patients with discrepancies between age and AMH levels in the early follicular phase prolonged protocol

**DOI:** 10.3389/fendo.2023.1098131

**Published:** 2023-03-08

**Authors:** Kexin Wang, Yichun Guan, Yuchao Zhang, Ruolin Jia, Shanshan Wu, Zhuolin Yao, Mingmei Zhang, Zhen Li

**Affiliations:** The Reproduction Center, The Third Affiliated Hospital of Zhengzhou University, Zhengzhou, China

**Keywords:** AMH, cumulative clinical pregnancy rate, cumulative live birth rate, maternal age, early follicular phase prolonged protocol

## Abstract

**Objective:**

To explore the cumulative outcomes and influencing factors of patients with discrepancies between age and Anti-Müllerian hormone (AMH) levels in the early follicular phase prolonged protocol.

**Methods:**

A total of 1282 cycles of in-vitro fertilization (IVF)/intracytoplasmic sperm injection (ICSI) assisted pregnancy with the early follicular phase prolonged protocol in the Reproductive Medicine Center of the Third Affiliated Hospital of Zhengzhou University from September 2015 to December 2020 were retrospectively analyzed. They were divided into the young low-AMH group (n=1076) and the older high-AMH group (n=206). The primary outcomes included cumulative clinical pregnancy rate (CCPR) and cumulative live birth rate (CLBR). Secondary outcomes included the number of oocytes retrieved, number of available embryos, clinical pregnancy rate (CPR), live birth rate (LBR), miscarriage rate (MR), pregnancy complications, and neonatal outcomes.

**Results:**

The CPR (68.7% vs. 59.4%) and the LBR (60.7% vs. 43.1%) in the young low-AMH group were higher than those in the older high-AMH group. In contrast, the number of oocytes retrieved (11 vs. 17), number of available embryos (5 vs. 8), and MR (10.6% vs. 18.3%) in the young low-AMH group were lower. There was no significant difference between the two groups in the CCPR, CLBR, pregnancy complications, and neonatal outcomes. Logistic regression analysis showed that infertility duration, basal follicle-stimulating hormone (FSH), and antral follicle count (AFC) correlated with CCPR, while maternal age, type of infertility, basal FSH, AFC, and infertility duration correlated with CLBR. The area under the receiver operating characteristic curves (ROC) curve for the combined model of infertility duration, AFC, and basal FSH to predict cumulative pregnancy was 0.629 (95%CI:0.592-0.666), while the combined model of maternal age, AFC, basal FSH, infertility duration, and type of infertility to predict cumulative live birth was 0.649 (95%CI:0.615-0.682).

**Conclusion:**

Although AMH levels are low by contrast, young patients have a favorable outcome after IVF/ICSI. In patients with discrepancies between age and AMH levels in the early follicular phase prolonged protocol, maternal age correlates better with cumulative live birth. The model that combines maternal age and other factors can help predict cumulative live birth, but its value is limited.

## 1 Introduction

Since the birth of the world’s first test-tube baby in 1978, assisted reproductive technology (ART) has been carried out for more than 40 years, bringing good news to millions of infertility patients (1). Controlled ovarian hyperstimulation (COH), as a critical part of ART, aims to obtain an appropriate number of mature follicles while avoiding complications such as ovarian hyperstimulation syndrome. Besides, the early follicular phase prolonged protocol has become one of the leading regimens of COH due to its advantages of high synchronization of follicular development, avoidance of early onset of Luteinizing hormone (LH) surge, improvement of endometrial receptivity, and increase of implantation rate and pregnancy rate (2).

In recent years, with the promotion of the single embryo transfer strategy and the wide application of Frozen-thawed embryo transfer (FET), the outcomes of not only the fresh embryo transfer cycles but also the FET cycles are included in the report of the outcomes of the ART (3). Previous studies have found that the CLBR represents all the live births after continuous ART treatment, which can better summarize the chances of live birth during the whole treatment period (4–6), suggesting that the cumulative outcomes of a complete assisted reproductive cycle (including fresh embryo transfer and FET), maybe more worthy of attention.

As an essential indicator of ovarian reserve, AMH plays a crucial role in reproductive development and regulation (7). Studies have shown that the relationship between AMH levels and maternal age shows a positive correlation trend before the age of 25 and gradually decreases with the increase of age from around 30 years old until menopause (8). However, due to individual heterogeneity, a certain number of women have discrepancies between age and AMH levels (some young women have a lower AMH level, while some older women have a higher AMH level) (9, 10). At present, there are few studies on these patients. This paper aims to explore the cumulative outcomes of patients with discrepancies between age and AMH levels in the early follicular phase prolonged protocol and analyze their influencing factors.

## 2 Materials and methods

### 2.1 Experimental design and subjects

This study retrospectively analyzed 1282 cycles of IVF/ICSI assisted pregnancy in the Reproductive Center of the Third Affiliated Hospital of Zhengzhou University from September 2015 to December 2020. Inclusion criteria: (1) the implementation of an IVF/ICSI cycle, (2) women aged between 20 and 48, (3) the COH regimen is the early follicular phase prolonged protocol. Exclusion criteria: (1) cycles of sperm donation or oocyte donation to assist pregnancy, (2) cycles of preimplantation genetic testing, (3) patients with abnormal uterine structure (such as the septate uterus), submucous uterine fibroids, endometriosis, adenomyosis, intrauterine adhesion, (4) Patients with a history of ovarian surgery (such as stripping of ovarian cysts), (5) chromosome abnormality of husband and wife, (6) abnormal data and data loss.

Patients were first grouped by age (< 35 years for young women and ≥35 years for older women). Then the AMH levels in the two groups were grouped according to the quartile method (P_0_-P_25_ were low-AMH groups, and P_75_-P_100_ were high-AMH groups) to obtain the final grouping, namely the young low-AMH group (n=1076) and the older high-AMH group (n=206).

### 2.2 Clinical protocol

In the early follicular phase prolonged protocol, 3.75 mg of gonadotrophin-releasing hormone agonist (GnRHa) (Triptorelin, Bofu-Yipusheng (Tianjin) Pharmaceutical Co Ltd, China) was injected into patients’ muscles on the 2nd to 3rd day of the menstrual cycle. Fasting blood was drawn after 28–30 days to measure FSH, luteinizing hormone (LH), estradiol, and progesterone levels, and follicular development and endometrial thickness (EMT) were monitored by transvaginal ultrasound. After reaching the pituitary down-regulation criteria (LH < 5 IU/L, estradiol < 50 ng/L, and EMT<5 mm), exogenous Gonadotropin (Gn) was administered for ovulation induction (75–300 IU/d). During the ovulation induction, the dosage was constantly adjusted according to the reactivity of the ovarian and hormone level. When the diameter of 2~3 dominant follicles is not less than ≥18 mm or the diameter of at least one dominant follicle is not less than ≥20 mm, Gn is stopped, and 250 ug of recombinant human chorionic gonadotropin (Azer, Merck Serono, the USA) trigger is intramuscularly injected. The oocytes retrieved under the guidance of vaginal ultrasound were performed 36–38 hours after the trigger. At the same time oocytes were retrieved, the husband shall conduct semen collection and semen optimization. Depending on the patient’s medical history, IVF or ICSI was administered. Corpus luteum support was given after oocytes retrieved.

### 2.3 AMH measurement

After excluding the use of contraceptives of the patients in the past three months, the serum AMH of the patient was collected within the 2nd - 4th days of the last menstrual cycle before ART treatment. Serum AMH is measured according to the standard operating procedures in the Endocrine laboratory through the method of the electrochemiluminescence immunoassay (ECLIA, Cobas 8000, Roche), meanwhile the internal quality control materials of Roche PreciControl AMH Plus were tested as samples on the Cobas system every day.

### 2.4 Embryo transfer and follow-up after transfer

Fresh embryo transfer: The cleavage-stage embryo was transferred 3 days after oocytes retrieved, or the blastula was transferred 5 days after oocytes retrieved. According to the recommendations of the American Reproductive Medicine Association and the China Reproductive Medicine Association, a maximum of two cleavage-stage embryos or one blastocyst can be transferred. Embryo transfer was performed under abdominal ultrasound guidance. Corpus luteum support was given after embryo transfer.

FET: According to the patient’s medical history, we formulated an individualized endometrial preparation plan, including natural cycle, hormone replacement cycle, downregulation combined with hormone replacement cycle and stimulation cycle. Routine corpus luteum support was given after the FET.

Follow-up after transfer: Serum β-human chorionic gonadotropin (β-hCG) was detected on the 14th day after embryo transfer, and the serum β-HCG > 50 IU/L was defined as positive. Transvaginal ultrasound 4–5 weeks after embryo transplantation showed that the patient with gestational sac was diagnosed as clinical pregnancy. Live births are defined as those with a 28-week gestation and delivery of a neonate with vital signs. CCPR per oocyte retrieved cycle = (the number of first clinical pregnancies in fresh and frozen cycles)/the number of oocytes retrieved cycles× 100%; The observation standard is to obtain at least one clinical pregnancy or use up all the embryos in this ovulation induction, and the observation time is 2 years (4, 11). CLBR per oocytes retrieved cycle = (the number of cycles in which live birth was first obtained during the fresh and frozen cycles)/the number of oocytes retrieved cycles × 100%; The end of observation was based on obtaining at least one live birth or using up all the embryos for this ovulation induction, and the observation time was 2 years (4, 11).

### 2.5 Statistical analysis

This study used SPSS 26.0 and R 3.6.3 for statistical analysis and visualization. After the measurement data were normalized by Kolmogorov-Smirnov test, the data were non-normally distributed, expressed as median (P_25_, P_75_), and inter-group comparison was conducted using Mann-Whitney U test. Enumeration data were expressed as rate (frequencies), and inter-group comparisons were performed using either the Chi-square test or the Fisher’s exact test. In order to exclude the influence of potential confounding factors, multivariate logistic regression was used to correct the basal characteristics between two groups and analyze the variables that may affect the cumulative outcomes, which was expressed by Odds Ratio (OR), adjusted Odds Ratio (aOR) and 95% Confidence Internal (CI). To assess performance of the resulting models we plotted ROC and calculated the area under the curve (AUC). P value < 0.05 was considered significant.

## 3 Results

### 3.1 Study population

**Figure 1:**
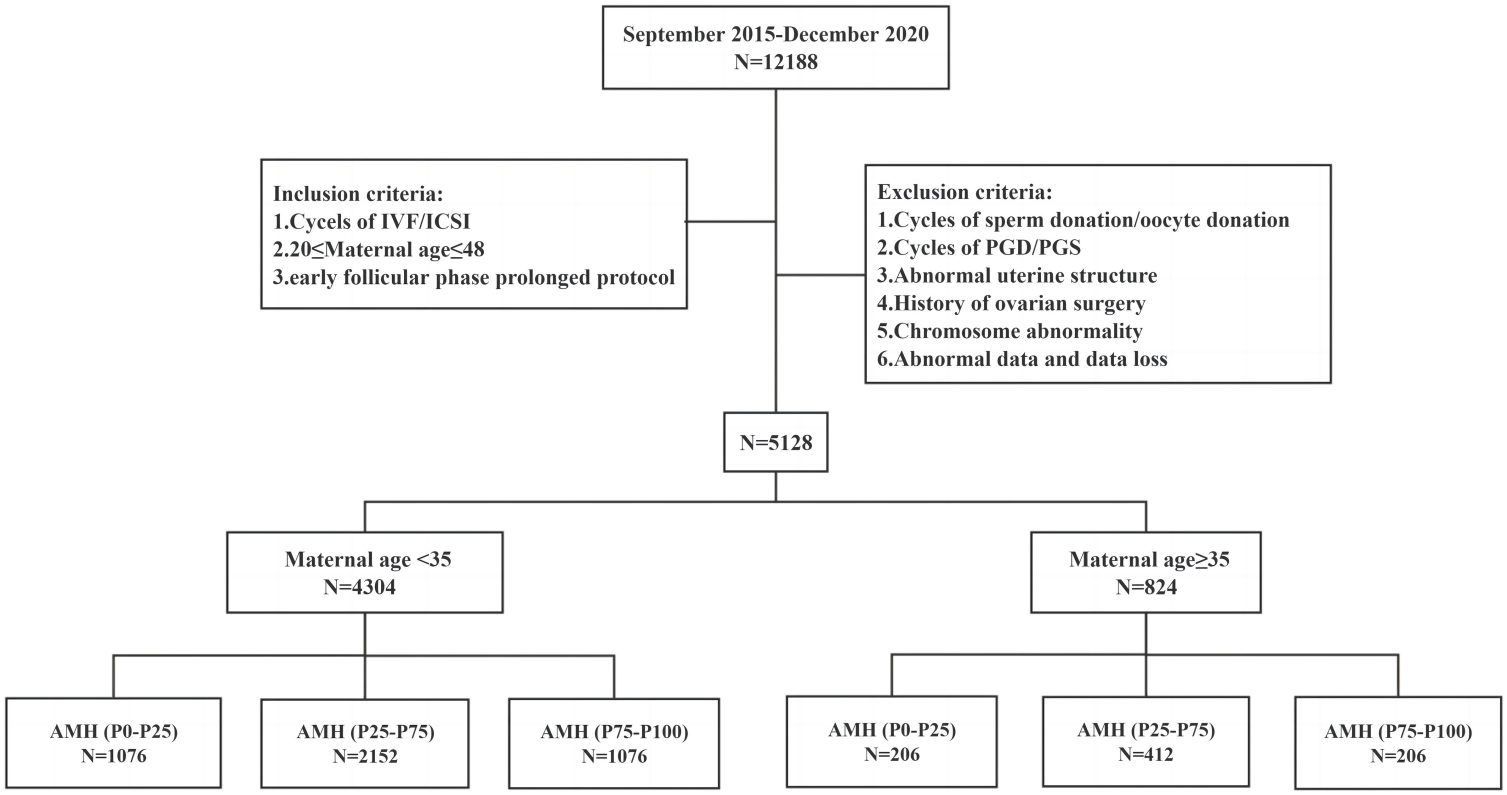
Flow chart of the study design.

This study retrospectively analyzed the 12188 cycles of ART from September 2015 to December 2020 in the Reproduction Center of the Third Affiliated Hospital of Zhengzhou University. After screening for inclusion and exclusion criteria, cycles were 4304 in the young group and 824 in the older group. In the young group, there were 1076 cycles of P_0_-P_25_ (1.63 pmol/L≤AMH<16.47 pmol/L), 2152 cycles of P_25_-P_75_ (16.47 pmol/L≤AMH<35.87 pmol/L), and 1076 cycles of P_75_-P_100_ (35.87 pmol/L≤AMH ≤ 164.22 pmol/L). In the older group, there were 206 cycles of P_0_-P_25_ (1.63 pmol/L≤AMH<13.64 pmol/L), 412 cycles of P_25_-P_75_ (13.64 pmol/L≤AMH<29.12 pmol/L) and 206 cycles of P_75_-P_100_ (29.12 pmol/L≤AMH ≤ 140.23 pmol/L). This study focused on the patients with discrepancies between age and AMH levels. The patients with AMH levels in P_0_–P_25_ in the young group (1076 cycles) were defined as the young low-AMH group, and the patients with AMH levels in P_75_-P_100_ in the older group (206 cycles) were defined as the older high-AMH group (**Figure 1**).

### 3.2 Characteristics of the study groups

**Table 1** shows the baseline characteristics of young low-AMH group patients and older high-AMH group patients. Significant differences were found between the two groups in maternal age (30.0 vs. 36.0), body mass index (BMI) (23.2 vs. 24.1), infertility duration (3 vs. 4), the proportion of secondary infertility (46.7% vs. 73.8%), AFC levels (14 vs. 22), and AMH levels (12.7 vs. 37.3), basal FSH levels (7.2 vs. 6.2), and initial dose of Gn (225.0 vs. 200.0) (P < 0.05). The percentages of ICSI (21.7% vs. 22.3%), total days of Gn (13 vs. 13), and EMT on the day of HCG (11.4 vs. 11.4) were consistent between the two groups (P > 0.05).

**Table 1:** Baseline and cycle characteristics between the two groups.

	young low-AMH group n=1076	older high-AMH group n=206	*P*
Meternal age (year)	30.0(28.0,32.0)	36.0(35.0,38.0)	<0.001
BMI (kg/m^2^)	23.2(21.0,25.6)	24.1(22.2,26.7)	<0.001
Infertility duration (year)	3(2,5)	4(2,7)	<0.001
Type of infertility (%)			<0.001
Primary infertility	53.3(574/1076)	26.2(54/206)	
Secondary infertility	46.7(502/1076)	73.8(152/206)	
Type of ART (%)			0.829
IVF	78.3(843/1076)	77.7(160/206)	
ICSI	21.7(233/1076)	22.3(46/206)	
Basal FSH (IU/L)	7.2(6.1,8.4)	6.2(5.4,7.2)	<0.001
AFC (No.)	14(11,17)	22(18,24)	<0.001
AMH (pmol/L)	12.7(10.2,14.6)	37.3(32.7,46.6)	<0.001
Initial dose of Gn (IU)	225.0(150.0,275.0)	200.0(150.0,225.0)	<0.001
Total days of Gn (day)	13(12,14)	13(12,14)	0.081
EMT on the day of HCG (mm)	11.4(9.9,13.0)	11.4(9.8,13.0)	0.632

### 3.3 Laboratory outcomes and ART outcomes

The laboratory outcomes and ART outcomes between the two groups are shown in **Table 2**. The number of oocytes retrieved (11 vs. 17), number of mature oocytes (9 vs. 14), number of two pronuclei (2PN) (7 vs. 11), number of available embryos (5 vs. 8), number of high-quality embryos (3 vs. 4) and MR (10.6% vs. 18.3%) in the young low-AMH group were lower than those in the older high-AMH group (P < 0.05). However, the CPR (68.7% vs. 59.4%) and LBR (60.7% vs. 43.1%) in the young low-AMH group were higher than that in the older high-AMH group (P < 0.05). There were no significant differences between the two groups in CCPR (77.9% vs. 82.5%) and CLBR (72.7% vs. 78.6%) (P > 0.05).

**Table 2:** Laboratory outcomes and ART outcomes between the two groups.

	young low-AMH group n=1076	older high-AMH group n=206	*P*
No.of oocytes retrieved	11(8,15)	17(13,22)	<0.001
No.of mature oocytes	9(6,13)	14(10,19)	<0.001
No.of 2PN	7(5,10)	11(7,15)	<0.001
No.of available embryos	5(3,8)	8(5,12)	<0.001
No.of high-quality embryos	3(1,5)	4(2,8)	<0.001
CPR (%)	68.7(998/1452)	59.4(190/320)	0.001
CCPR (%)	77.9(838/1076)	82.5(170/206)	0.136
LBR (%)	60.7(881/1452)	43.1(138/320)	<0.001
CLBR (%)	72.7(782/1076)	78.6(162/206)	0.075
MR (%)	10.6(89/838)	18.3(32/175)	0.004

### 3.4 Complications of pregnancy and neonatal outcomes

**Table 3** shows the pregnancy complications and neonatal outcomes between the two groups. There was no significant difference in gestational hypertension (3.5% vs. 3.5%), gestational diabetes (5.4% vs. 3.5%), hypothyroidism during pregnancy (0.2% vs. 0%), anemia in pregnancy (0.4% vs. 0%), fetal distress (0.1% vs. 0%), premature rupture of membranes (2.3% vs. 1.8%), preterm delivery (12.1% vs. 14.8%), full-term delivery (86.3% vs. 85.2%), post-term delivery (1.5% vs. 0%), neonatal birthweight (3280 vs. 3400), low birthweight neonates (10.9% vs. 12.3%) and macrosomia(12.5% vs. 14.2%).

**Table 3:** Comparison of pregnancy complications and neonatal outcomes between the two groups.

	young low-AMH group n=1076	older high-AMH group n=206	*P*
Gestational hypertension (%)	3.5(29/838)	3.5(6/170)	0.964
Gestational diabetes (%)	5.4(45/838)	3.5(6/170)	0.318
Hypothyroidism during pregnancy (%)	0.2(2/838)	0(0/170)	0.524
Anemia in pregnancy (%)	0.4(3/838)	0(0/170)	0.435
Fetal distress (%)	0.1(1/838)	0(0/170)	0.652
Premature rupture of membranes (%)	2.3(19/838)	1.8(3/170)	0.683
Type of delivery (%)			0.205
premature delivery	12.1(95/782)	14.8(24/162)	
full-term delivery	86.3(675/782)	85.2(138/162)	
post-term delivery	1.5(12/782)	0(0/162)	
Neonatal birthweight (g)	3280(2900,3600)	3400(2995,3800)	0.089
Low birthweight (%)	10.9(85/782)	12.3(20/162)	0.587
Macrosomia (%)	12.5(98/782)	14.2(23/162)	0.564

### 3.5 Multivariate logistic regression analysis

In order to analyze the influencing factors of cumulative outcomes in patients with discrepancies between age and AMH levels, we selected the maternal age, the BMI, the infertility duration, type of infertility, type of ART, basal FSH, AMH, and AFC as independent variables, and the cumulative outcomes as the dependent variable for multivariate Logistic regression analysis, and used a backward elimination method to create the best fitting regression model (**Table 4**). The results showed that in patients with discrepancies between age and AMH levels, the factor significantly associated with the CCPR was infertility duration (aOR: 0.94; 95%CI: 0.89-0.99), AFC (aOR: 1.06; 95%CI: 1.04-1.09) and basal FSH (aOR: 0.91; 95%CI: 0.86-0.97); the factor significantly associated with the CLBR was the maternal age (aOR: 0.96; 95%CI: 0.92-0.99), AFC (aOR: 1.07; 95%CI: 1.05-1.10), basal FSH (aOR: 0.91; 95%CI: 0.86-0.97), infertility duration (aOR: 0.92; 95%CI: 0.88-0.97), and type of infertility (aOR: 1.41; 95%CI: 1.07-1.85). However, AMH, BMI, and type of ART have no obvious correlation in the cumulative outcomes of patients with discrepancies between age and AMH levels.

**Table 4:** Logistic regression analysis of cumulative outcomes and influencing factors of patients with discrepancies between age and AMH levels.

	CCPR				CLBR			
	OR	aOR	95%CI	*P*	OR	aOR	95%CI	*P*
Meternal age (year)	0.99	0.96	0.92-1.01	0.076	0.99	0.96	0.92-0.99	<0.037
Infertility duration (year)	0.93	0.94	0.89-0.99	0.011	0.92	0.92	0.88-0.97	<0.001
Type of infertility (%)								
Primary infertility	1	1	1		1	1	1	
Secondary infertility	1.39	1.29	0.96-1.73	0.089	1.46	1.41	1.07-1.85	0.015
Type of ART (%)								
IVF	1	1	1		1	1	1	
ICSI	0.71	0.74	0.53-1.03	0.071	0.75	0.78	0.57-1.06	0.111
BMI (kg/m^2^)	1.01	0.99	0.95-1.03	0.561	1.01	0.99	0.95-1.02	0.434
Basal FSH (IU/L)	0.88	0.91	0.86-0.97	0.004	0.89	0.91	0.86-0.97	0.003
AMH (pmol/L)	1.01	1.01	0.99-1.02	0.458	1.02	1.01	0.99-1.03	0.175
AFC (No.)	1.07	1.06	1.04-1.09	<0.001	1.07	1.07	1.05-1.10	<0.001

### 3.6 Receiver operating characteristic curves

**Figure 2:**
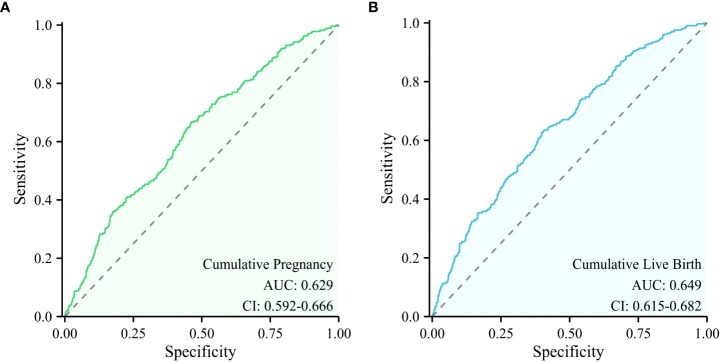
ROC curve of the models with the highest predictive value for cumulative pregnancy and cumulative live birth. **(A)** A model for predicting cumulative pregnancy, **(B)** A model for predicting cumulative live birth.

The backward elimination method logistic regression model retained infertility duration, basal FSH and AFC as significant independent predictors of cumulative pregnancy, while the independent predictors of cumulative live birth were maternal age, basal FSH, AFC, type of infertility, and infertility duration. Different combinations of these independent predictors were used to construct models to predict cumulative pregnancy and cumulative live birth, respectively, and ROC was used to assess the predictive value of these models (**Figure 2**). Among many models constructed, the model with the highest predictive value of cumulative pregnancy is the model constructed by AFC level, basal FSH level, and infertility duration, and the AUC is 0.629(0.592-0.666), while the highest predictive value of cumulative live birth is the model constructed by maternal age, AFC level, basal FSH level, infertility duration and type of infertility, and its AUC is 0.649(0.615-0.682).

## 4 Discussion

This study found that among patients with discrepancies between age and AMH levels, patients with high AMH levels had a better ovarian response, but the outcomes of ART were not the same. Young low-AMH group patients had better CPR and LBR than older high-AMH group patients, and there were no significant differences in CCPR or CLBR between the two groups. In addition, maternal age correlated better with cumulative live birth than AMH levels in patients with discrepancies between age and AMH levels. Moreover, the predictive value of models constructed with a combination of multiple hormones and clinical parameters for cumulative outcomes was higher compared with a single biomarker but still at a lower level.

Serum AMH levels are considered an essential factor in predicting ovarian response to COH due to the ability to reflect the size of the primordial follicle pool and oocyte quality (12). Previous studies have found a strong positive correlation between serum AMH levels and the number of oocytes retrieved (12–14). Seifer et al. found that the patients with the number of oocytes retrieved ≥ 11 had a higher AMH levels than those with number of oocytes retrieved ≤6 (15). Consistent with Seifer’s study, the number of oocytes retrieved in our study was significantly higher in the high-AMH group than in the low-AMH group (17 vs. 11). However, this does not mean that the number of oocytes retrieved in our study was unsatisfactory in patients with low and middle AMH levels. Wu et al. observed that there were some patients whose number of oocytes retrieved was ideal despite the low level of AMH (16). In the study of Wu et al., compared to the control group, patients with low AMH levels but the ideal number of oocytes retrieved were younger and had higher basal FSH levels, as confirmed in our study. This may be because, on the one hand, high-level FSH can inhibit the expression of AMH induced by growth and differentiation factor 9 (GDF9) and bone morphogenetic protein 15 (BMP15) through the protein kinase A pathway, and on the other hand, due to the reduction of AMH levels, leading to a weakened inhibition of primordial follicles by AMH, thus stimulating and recruiting more follicles from the follicle pool into the development stage (16–18).

Although older patients had higher AMH levels than young patients, their CPR and LBR were significantly lower than those in the young group. This reminds us that although a high level of AMH predicts better ovarian reserve, we cannot ignore the reproductive aging brought by aging (19). In a cohort study, Ntostis found that, compared with young patients, the expression of mitochondrial-related genes in metaphase II oocytes in older patients was imbalanced, leading to the aging of mitochondrial structure and functions (19). Due to the significant decrease in mitochondrial respiratory capacity in aged oocytes, adenosine triphosphate (ATP) production decreases, which eventually leads to a decrease in the metabolic activity of oocytes, affecting fertilization, embryo development, and implantation (20–22). In addition, high expression of family members of the peroxiredoxin (PRDX) gene (including PRDX1, PRDX2, PRDX4, and PRDX6) in young patients indicates that oocytes in the older group show reduced protection against oxidative stress and are vulnerable to oxidative damage thereby reducing oocyte quality and fertility (23, 24). In addition to maternal age, some specific diseases will also have a particular limitation on ovarian stimulation, affecting ART outcomes. In our study, the serum AMH of patients in the older group was at a high level, and polycystic ovary syndrome (PCOS) patients accounted for a certain proportion. However, PCOS patients often suffered from oocyte quality injury. Previous studies have found that when the serum AMH levels of PCOS patients exceeded the threshold, their ovarian reactivity would decrease, which made these patients less responsive to low-dose drugs to promote ovulation, and ultimately affected their outcomes of ART (25). Therefore, more and more studies recommend the application of insulin sensitizers to improve the oocyte quality and reduce the dose of Gn when ovarian stimulation is performed on patients with PCOS (26). In addition, endometriosis has been proven to reduce ovarian reserve and oocyte quality, thus affecting ovarian stimulation (27). However, because endometriosis impacts the pelvic environment, it was not included in this study.

Of course, the increase in maternal age brings not only the decline of oocyte quality but also the impact on the MR of patients. Our study found that the MR in older high-AMH group patients was significantly higher than in young low-AMH group patients. Although studies have linked serum AMH levels to MR after IVF-ET, the study by Grande et al. found no increase in MR in patients with low AMH levels (28, 29). This means that in patients with discrepancies between age and AMH levels, maternal age has a greater effect on the MR than serum AMH levels, as was consistent with Zhang et al. (10).

Given that multiple transplants of the same patient may have been performed in our study, the evaluation of clinical outcomes is of limited value, so the evaluation of cumulative outcomes is of greater value for this study (30–32). When Zhang et al. studied IVF outcomes in patients with discrepancies between age and AMH levels, they found that there was no significant difference in CCPR and CLBR between older high-AMH group patients and young low-AMH group patients (10). The potential reason may be that the increased number of transplants improves the cumulative outcomes. Chen et al. found in their study that the CLBR showed a significant upward trend with the prolongation of time or the increase in the number of FET (33). This suggests that, although older high-AMH group patients may have decreased CPR and LBR due to decreased oocyte quality as they get older, with the increase in the number of transplantation cycles, they may achieve cumulative outcomes comparable to those in young patients.

Maternal age and serum AMH levels have been previously considered predictors of CLBR after IVF/ICSI (34, 35). However, our study shows maternal age is more critical than AMH levels in predicting cumulative live birth in patients with discrepancies between age and AMH levels. A retrospective analysis by Tal et al. found that in patients with decreased ovarian reserve, both serum AMH levels and maternal age independently provide predictive information for CLBR (36). Nevertheless, whether serum AMH levels can be used as a marker to predict the outcomes of IVF/ICSI is still controversial (37). Different from Tal, Li et al. found that while AMH was predictive of CLBR, after maternal age adjustment, AMH was not a separate predictor of CLBR (38). In a similar study of patients with discrepancies between age and AMH levels, AMH was found to have poor predictors of CLBR in young low-AMH patients and in older high-AMH patients (10). This is consistent with our study, which means that serum AMH levels can only partially assist in predicting the cumulative outcomes for patients with discrepancies between age and AMH levels. The reason for the poor predictability of AMH levels may be closely related to the choice of the research population. Serum AMH levels in women without PCOS are often considered to be positively correlated with Gn-stimulated ovarian response (15). However, this does not mean we can take the serum AMH levels measured in the general population as the standard to judge natural fertility. Xi et al. found that PCOS women with high AMH levels had lower CPR and embryo implantation rates (39). Further study found that the ovaries of PCOS patients with high AMH levels showed resistance to Gn stimulation. In exploring the effect of serum AMH levels on ovarian responsiveness in women with PCOS, Amer et al. found that ovarian responsiveness was reduced in women with PCOS when AMH levels were above a threshold (4.7 ng/ml or 33.6 pmol/L) (40). Therefore, elevated AMH levels may not lead to good pregnancy outcomes for PCOS women with high AMH levels. At the same time, it also reminds us that women with PCOS clinically facing high AMH levels may need to be given higher doses of Gn to obtain the desired pregnancy outcomes.

In our study, the independent predictors of cumulative live birth and cumulative pregnancy were combined to construct different models, and the predictive value of these models was compared. We find that the predicted value of the model with more factors is higher than that of the model with fewer factors. Balachandren found that the combined model of maternal age, FSH category, and AMH category had good predictive value for CLBR, with the AUC of the ROC being 0.68(95%CI:0.63-0.73) (41). In our study, the area under the ROC curve of the optimal model constructed for predicting cumulative live birth was 0.649 (0.615–0.682). However, although this model has the highest predictive value among all the models we constructed, its predictive accuracy still relatively low. This suggests that we consider this multifactorial disease comprehensively and not judge the disease only by some indicators or parameters.

This study fills the gap in the field of cumulative outcomes and influencing factors for patients with discrepancies between age and AMH levels in early follicular phase prolonged protocol. At the same time, we constructed multiple models for ART outcomes and found that for multifactorial disease, a combination of multiple common predictors in reproductive centers is often required to estimate the individual probability of pregnancy or live birth after IVF/ICSI cycles. Our study also has some limitations. This study is a single-center study. On the one hand, the study population may be specific, and on the other hand, the study sample size may need to be increased compared with multi-center studies. In addition, only patients with early follicular phase prolonged protocol were selected in this study. Whether the protocol change affects ovarian stimulation and outcomes is still unclear, and the extrapolation is relatively insufficient. Therefore, the results of this study still need to be verified by multiple centers.

## 5 Conclusions

In conclusion, despite relatively low levels of AMH, young patients have favorable outcomes after IVF/ICSI. In patients with discrepancies between age and AMH levels in early follicular phase prolonged protocol, maternal age correlates better with outcomes than AMH levels. In addition, the multi-factor combined model is more valuable in predicting the cumulative outcomes of patients with discrepancies between age and AMH levels in early follicular phase prolonged protocol than the model with few factors.

## Data availability statement

The raw data supporting the conclusions of this article will be made available by the authors, without undue reservation.

## Ethics statement

The studies involving human participants were reviewed and approved by This study has been approved by the Ethics Committee of the Third Affiliated Hospital of Zhengzhou University with the approval number of 2022-154-01. Written informed consent for participation was not required for this study in accordance with the national legislation and the institutional requirements. Written informed consent was not obtained from the individual(s) for the publication of any potentially identifiable images or data included in this article.

## Author contributions

KW and ZL designed the study. ZY and MZ extracted the data. RJ and SW reviewed the data. KW were involved analyzed the data and drafted this article. YG and YZ revised this article. All authors contributed to the article and approved the submitted version.
